# Clinical Parameters to Predict Future Clinical Disease Activity After Treatment Change to Higher-Dose Subcutaneous Interferon Beta-1a From Other Platform Injectables in Patients With Relapsing-Remitting Multiple Sclerosis

**DOI:** 10.3389/fneur.2020.00944

**Published:** 2020-09-02

**Authors:** Martina Novotna, Ales Tvaroh, Jan Mares

**Affiliations:** ^1^Department of Neurology and Center of Clinical Neuroscience, General University Hospital, Charles University, Prague, Czechia; ^2^Merck spol. s r.o, Prague, Czechia; ^3^Department of Neurology, Krajska zdravotni, a.s.—Nemocnice Teplice, o.z., Teplice, Czechia; ^4^Department of Neurology, MS Center, Faculty Hospital, Palacky University, Olomouc, Czechia

**Keywords:** multiple sclerosis, relapse, progression, disease activity, interferon beta

## Abstract

**Objective:** To identify predictors of clinical disease activity after treatment change to higher-dose interferon beta-1a in relapsing-remitting multiple sclerosis (MS).

**Methods:** This was a retrospective-prospective observational multicenter study. We enrolled patients with at least one relapse on platform injectable therapy who were changed to 44 μg interferon beta-1a. Our primary endpoint was the clinical disease activity-free (cDAF) status at 6, 12, 18, and 24 months. Secondary endponts included relapse-free status and disability progression-free status at different timepoints. The primary predictor of interest was the monosymptomatic vs. polysymptomatic index relapse, based on the number of affected functional systems from the Kurtzke scale during the last relapse prior to baseline. Other secondary predictors of clinical disease activity were analyzed based on different demographic and relapse characteristics. Kaplan-Meier estimates of the cumulative probability of remaining in cDAF status were performed. The time to clinical disease activity was compared between groups using univariate Kaplan-Meier analysis and multivariate Cox regression. Multivariate analyses were processed in the form of CART (Classification & Regression Trees).

**Results:** A total of 300 patients entered the study; 233 (77.7%) of them completed the 24-month study period and 67 patients (22.3%) terminated early. The proportion of patients in cDAF status was 84.7, 69.5, 57.5, and 54.2% at 6, 12, 18, and 24 months. After 2 years of follow-up, 55.9% of patients remained relapse-free and 87.8% of patients remained disability progression-free. At all timepoints, the polysymptomatic index relapse was the most significant predictor of clinical disease activity of all studied variables. Hazard ratio of cDAF status for patients with monosymptomatic vs. polysymptomatic index relapse was 1.94 (95% CI 1.38–2.73). CART analyses also confirmed the polysymptomatic index relapse being the strongest predictor of clinical disease activity, followed by higher number of pre-baseline relapses with the most significant effect in the monosymptomatic index relapse group. The next strongest predictors of clinical disease activity were cerebellar syndrome as the most disabled Kurtzke functional system for the monosymptomatic relapse group, and age at first MS symptom ≥ 45 for the polysymptomatic relapse group.

**Conclusions:** Patients with a polysymptomatic index relapse and/or higher number of relapses within 2 years prior to baseline are at high risk of clinical disease activity, despite treatment change to higher-dose interferon beta-1a from other platform injectable therapy.

**Trial registration:** State Institute of Drug Control (SUKL), URL: http://www.sukl.eu/modules/nps/index.php?h=study&a=detail&id=958&lang=2, registration number 1205090000.

## Introduction

The primary goal of disease-modifying treatments (DMTs) in multiple sclerosis (MS) is to change the natural course of the disease by reducing the number and severity of relapses and by preventing progression of disability. Injectable first line DMTs have proven to reduce frequency of relapses by about one third (1–3). They also reduced the severity of relapses and had a significant effect on magnetic resonance imaging (MRI) measures of disease activity. The appropriate dose of interferons continues to be debated. Results of an extension of the pivotal PRISMS trial demonstrated a continued benefit in patients originally randomized to 44 μg dose of interferon beta-1a (IFN β-1a) compared with those receiving 22 μg dose or whose treatment had been delayed (4–7). Results of a 15-year follow-up of the PRISMS trial suggest that a higher cumulative exposure to subcutaneous (sc) IFN β-1a may be associated with better clinical outcomes (8). In addition, the EVIDENCE (Evidence of Interferon Dose-response-European North American Comparative Efficacy) trial comparing relative efficacy of two different dosing regimens of IFN β-1a showed significant benefit on relapse rate and MRI outcome measures of higher-dose (44 μg) three times a week sc interferon therapy, compared with low-dose (30 μg) intramuscular (im) weekly interferon therapy after 48 weeks, and these benefits were sustained for up to 64 weeks (9–11). In a cross-over extension of the EVIDENCE trial patients receiving IFN β-1a improved on clinical and MRI outcome measures after they changed from 30 μg once-weekly to 44 μg three times a week (tiw) (10, 12). Also, in the INCOMIN (Independent Comparison of Interferon) study, 250 μg of interferon beta-1b (IFN β-1b) every other day has been shown to have greater clinical and MRI benefits in RRMS patients compared with 30 μg of IFN β-1a once weekly (13).

Indirect comparison of large randomized trials suggests a higher efficacy on relapses of higher-dose sc IFN β-1a (44 μg) tiw compared to im IFN β-1a, sc IFN β-1a 22 μg tiwor glatiramer acetate (GA) (14). Before the era of new high-efficacy drugs, the International Working Group for Treatment Optimization in MS recommended switching to high-dose IFN beta in patients who have suboptimal responses to low-dose IFN beta or GA (15). A German prospective study by Masri et al. (16) followed patients with insufficient response to first-line DMTs using the three-scale “analog” model. In this model earlier introduced by Bashir (17), each of the three critical indicators, relapses, disability progression and MRI, are rated on an analog scale of none or 0, notable, worrisome, and actionable. Optimizing treatment from another long-term DMT to sc IFN β-1a (44 μg tiw) resulted in a clinical and radiological stabilization of the disease in RRMS patients (16).

Clinical parameters to predict response to interferons in RRMS patients have also been studied with variable results, mainly due to lack of standardized definition of treatment response. However, not enough evidence-based information can be found on the efficacy of IFN β in patients with RRMS based on the different relapse phenotypes. Studies in patients with clinically isolated syndrome (CIS) have described efficacy of IFN β according to the phenotype of presenting clinical event and number of brain MRI lesions at baseline (monofocal vs. multifocal). The *post-hoc* analyses of the CHAMPS (Controlled High-Risk Subjects Avonex Multiple Sclerosis Prevention Study; 30 μg im once weekly) (18, 19) and BENEFIT (Betaferon/Betaseron in Newly Emerging MS for Initial Treatment; 250 μg sc every other day) (20, 21) studies concluded that IFN β was beneficial in patients with CIS regardless of phenotype of presenting clinical event, yet, IFN β efficacy was more pronounced in patients presenting with monofocal vs. multifocal events. In the REFLEX (REbif FLEXible dosing in early MS; sc IFN β-1a 22 μg tiw vs. once a week vs. placebo) study the treatment effect with the three times a week regimen was greater than that with the once a week regimen in the group of patients with multifocal presentation (22, 23). Likewise, in the 3- and 5-year extension of the REFLEX study (REFLEXION) there was a significantly increased risk of conversion to clinically definite MS in patients with multifocal vs. monofocal presentation (24).

Nevertheless, the above mentioned studies were performed in CIS population using IFN β as the first-line therapy in treatment-naïve patients. Data addressing the relationship between number of neurological functional systems involved in a relapse (monosymptomatic vs. polysymptomatic) in patients with RRMS treated with IFN β as an “escalation” therapy have not yet been published. Also, the term monosymptomatic/polysymptomatic cannot be equated to monofocal/multifocal presentation.

The original study by Runmarker and Andersen assessing prognostic factors in MS incidence cohort with 25-years of follow up found that having had symptoms from more than one region at the last bout was significantly unfavorable 15 and 25 years after onset. This study has further demonstrated that symptoms from afferent fibers were prognostically significantly better than symptoms from efferent fibers or mixed symptoms. The occurrence of polyregional symptoms at the last bout remained a significant unfavorable factor not only during the first 5 years of disease, but also for the long-term prognosis (25). Identifying prognostic markers of an individual's clinical course and responsiveness to a therapeutic modality, and thus moving a treatment decision-making process toward more individually-tailored medicines, is of inestimable importance with expanding treatment options in MS.

The aim of our study was to investigate whether number of functional systems involved in an index relapse (monosymptomatic vs. polysymptomatic), and possibly other clinical and demographic characteristics, may impact the clinical disease activity after treatment change from first-line injectable DMTs to higher dose of IFN beta-1a.

## Methods

### Study Design and Population

This was an observational, non-interventional, retrospective-prospective study, carried out at 16 MS centers in the Czech Republic and Slovakia, with enrollment during years 2012–2013. Adult RRMS patients fulfilling the 2010 revised McDonald diagnostic criteria (26) who experienced at least one relapse on a platform injectable therapy (im IFN β-1a, sc IFN β-1a 22 μg tiw, sc IFN β-1b or GA) and underwent treatment change to sc IFN β-1a 44 μg tiw not longer than in previous 12 months were eligible for the study. None of these patients has received other immunosuppressive therapy such as azathioprine or methotrexate within the last 3 months prior to baseline. Maximum baseline Kurtzke Expanded Disability Status Scale (EDSS) (27) score was 6.5. The final decision on treatment management had been made by the treating physician together with the patient, according to that patient's status, treatment guidelines at the time of study enrollment and standard clinical practice. All patients signed a written informed consent prior to any study-related assessments and the study was approved by each center's local Institutional Review Board.

### Data Collection and Assessment

The data were recorded in an observational manner, as a part of routine clinical practice. To assure quality of the analyzed data, only information from MS centers were used, where patients are followed by MS specialists periodically every 3 or 6 months as a part of routine clinical practice.

The study data were assembled in terms of two methods: demographic, overall health status and prior disease course data were collected retrospectively when a patient was enrolled in the study. Then, actual clinical status data were collected prospectively in the given timepoints during routine visits. The date of treatment change to IFN β-1a 44 μg was set as the baseline. According to the inclusion criteria, baseline procedures and retrospective data collection could have been performed between baseline and month 12 visit, usually during a routine visit when a decision to enter into the study was taken. Data assessment of month 6 visit therefore could have been entered into the electronic case report form from patient's medical records retrospectively, if treatment change has occurred more than 6 months prior to entering the study and further data were collected prospectively.

Duration of MS was derived from the time of the first clinical manifestation of the disease in the patient's record. Disability was assessed using the Kurtzke Expanded Disability Status Scale (EDSS) (27). For all events, including clinical relapses, disability progression, laboratory assessments, adverse events, adverse drug reactions, or any other new symptoms, the actual date of event onset was recorded.

The assessments related to the study endpoints were performed at the baseline, month 6, 12, 18, and 24; and at early termination visit, if applicable. The month 6, 12, 18, and 24 visits took place at 6, 12, 18, and 24th month, respectively, with an acceptable time-window of ± 4 weeks for each visit. However, if patient's assessment on the actual visit showed EDSS progression by at least 1.0 point in comparison to the baseline EDSS score, additional “EDSS progression confirmation visit” was scheduled. This visit took place 3 months after the study visit, when the occurrence of EDSS progression was assessed, to confirm whether the EDSS progression was sustained for at least 3 months or not.

Safety assessments were performed during routine visits, including laboratory tests according to clinical standards (liver function and blood count monitoring every 3 months during the first year of IFN treatment and later every 6 months, thyroid function monitoring before IFN treatment and later every 12 months). Each suspected adverse event and adverse drug reaction occurring during the study, whether serious or not, must have been recorded in the report forms, including its detailed description and actions taken with the study drug, required treatment and outcome.

### Study Endpoints

The primary endpoint of the study was the clinical disease activity-free status at 6, 12, 18, and 24 months. Clinical disease activity-free (cDAF) status was defined as the absence of both relapse(s) and disability progression. A qualifying relapse was defined as a sudden onset of new neurological symptoms or a sudden worsening of current symptoms (preceded by at least 30 days of clinical stability or improvement), lasting for longer than 24 h and leading to a 2-grade increase in one or more functional system scores from the Kurtzke functional scale (KFS) (27) or a 1-grade increase in two or more KFSs, excluding changes in bowel/bladder or cerebral/mental function, without clear presence of other causes (e.g., concurrent fever or infection). Sustained disability progression was defined as an increase in EDSS score by at least 1.0 point assessed on the actual visit, compared to the baseline EDSS score, and confirmed on the “EDSS confirmation” visit 3 months later. Secondary endpoints included the relapse-free status and sustained disability progression-free status at 6, 12, 18, and 24 months.

### Baseline Predictive Factors

The last relapse prior to baseline was considered the index relapse. The monosymptomatic vs. polysymptomatic index relapse phenotype was the primary predictor of interest. Monosymptomatic index relapse represents the last pre-baseline relapse affecting only one of the following KFSs: pyramidal, cerebellar, brainstem, sensory/pain or visual/optic (which could be accompanied by worsening of bowel/bladder and/or cerebral/mental functions), as opposed to a polysymptomatic relapse, with neurological worsening in at least two of the above mentioned KFSs.

To identify other baseline prognostic factors associated with cDAF status, we also performed analyses of secondary predictors of interest based on different demographic and relapse features and type and severity of neurological disability. These were: sex, age at first MS symptom, age at baseline, dominant symptom of the index relapse, relapse count in the 2 years prior to baseline, baseline EDSS score, the most affected baseline KFS and type of DMT prior to baseline. The KFS with the highest score was assigned the most affected KFS category, except for the value 9 (unknown). If two or more KFSs had equally high scores during examination, the final decision on the most affected KFS was arbitrated by the investigator, taking into account the patient's symptoms. Because of the low frequency of brainstem, bowel/bladder, or cerebral/mental “most affected baseline KFS,” these categories were combined into “other”.

### Data Analyses

A total of 300 subjects were recruited, assuming a dropout rate of around 15%. Thus, 255 subjects were expected to complete the follow-up period as per protocol. With this number of subjects, the proportion of individuals in the cDAF status at a specific time-point could be estimated with a maximum 95% confidence interval (CI) width of ± 7.5%. Assuming that 50% (*n* = 127) of subjects are in the monosymptomatic index relapse group, then the proportion of individuals in the cDAF status could be estimated with a maximum 95% CI width of ± 10.6%.

All data were summarized descriptively using mean, standard deviation (SD), median, minimum and maximum, or absolute (counts) and relative (percentages) frequencies, as appropriate. Baseline characteristics were compared between the monosymptomatic and polysymptomatic index relapse group using parametric ANOVA test (*T-*test) for variables with expected normal distribution (age), and non-parametric tests (Wilcoxon test or median test, as appropriate) for variables with non-normal data distribution (disease duration, EDSS, number of relapses, duration of DMT). The categorical variables were evaluated using the chi-square test (sex, the most disabled KFS, DMT prior to baseline).

Kaplan-Meier survival estimates of a cumulative probability of remaining free from clinical disease activity were performed. Patients were censored at the time of their last assessment if they terminated early or if the endpoint had not been reached by the end of the observation period. The impact of individual predictors of post-baseline clinical disease activity was tested using the log-rank test. Hazard ratios (HRs) and 95% confidence intervals (CIs) were estimated using Cox regression model. Cox regression models were used for continuous outcome data (time to event data) and for multivariate analyses.

To give comprehensive overview of risk factors, their linkages and probability of cDAF for each factor combination, the multivariate analyses were processed in the form of CART (Classification & Regression Trees) (28–32). All study variables entered into CART as explanatory factors, with all possible combinations of risk groups. Specifically, the multivariate analyses included: sex, whether the index relapse was polysymptomatic, number of relapses 2 years prior to baseline, dominant symptom of the index relapse, EDSS, the most disabled KFS, type of DMT prior to baseline, age at baseline and either disease duration (Model A), or age at first MS symptom (Model B). Since both variables “disease duration” and “age at first MS symptom” are a linear difference in relation to the variable “age at baseline,” they were not included to the multivariate analyses at the same time but were analyzed separately in two models. For age-related variables we have tested different cut offs (age quantiles).

In each point of the regression tree we searched for the next statistically strongest factor using various statistical methods (including the Cox regression model—stepwise regression and score test). This procedure was repeated for each of the already defined points (predictors of clinical disease activity). If no factor was found at the end of a given branch, the testing within this branch was terminated. The statistical significance of a given factor was further confirmed by the log rank test. Final CART therefore included only those factors which showed statistical significance in the regression analysis and also when using the log-rank test. The minimum number of patients in each branch of the regression tree was set to 5. *P* < 0.05 were considered statistically significant. SAS software (9.4; SAS Institute, Cary, NC, USA) and SW Statistica (StatSoft, Inc., Tulsa, OK, USA) were used for all data analyses.

## Results

A total of 300 patients from 11 Czech and 5 Slovak MS centers entered the study; 233 (77.7%) of them completed the 24-month study period and 67 patients (22.3%) terminated early. The median follow-up period was 2.1 years for the total population; 2.2 years for the patients who completed the study period; and 1.6 years for those who terminated early. Reasons for early termination included insufficient efficacy (13%), safety/tolerability reasons (4%), voluntary withdrawal (2.7%), confirmed pregnancy (2%), or other reasons (0.7%).

Baseline demographic and disease characteristics (Table 1) reflect a typical population of patients with RRMS. 75% of the study population were female (*n* = 224), mean age at baseline was 38.1 years. All patients were of Caucasian descent. Of the 300 patients analyzed in the study there were 160 (53.3%) patients with a monosymptomatic index relapse (presenting with only one affected KFS) and 140 (46.7%) patients with a polysymptomatic index relapse (presenting with more than one affected KFSs). The patients in the monosymptomatic relapse group were significantly younger at baseline and at their first MS symptom, had lower EDSS, fewer relapses in the 2 years prior to baseline and longer duration of DMT use compared to patients in the polysymptomatic relapse group (Table 1). The distribution of the most affected KFS and dominant symptom of the index relapse also differed significantly between the mono- and polysymptomatic relapse group, with visual/optic functions more commonly affected in patients with a monosymptomatic index relapse, and pyramidal functions more commonly affected in patients with a polysymptomatic index relapse. There was no significant difference in sex, disease duration, or type of DMT prior to baseline between the two index relapse groups.

**Table 1:** Baseline demographic and clinical characteristics.

	**Total (*n =* 300)**	**Monosymptomatic relapse group (*n =* 160)**	**Polysymptomatic relapse group (*n =* 140)**	***p-value***
**Baseline characteristics**				
*Age at baseline, yrs (mean ± SD)*	38.1 ± 10.1	36.4 ± 10.0	40.0 ± 10.0	*0.002^a^*
*Female, n (%)*	224 (75%)	123 (76.9%)	101 (72.1%)	*0.35^b^*
*Age at first MS symptom, yrs (mean ± SD)*	30.3 ± 9.7	28.4 ± 9.7	32.5 ± 9.2	*<0.001^a^*
*Disease duration, yrs*				
Median (range)	6.7 (0.2–30.3)	6.7 (0.2–30.3)	6.7 (0.5–24.6)	*0.73^c^*
*EDSS*				
Median (range)	2.5 (0–6.5)	2.0 (1.0–6.0)	3.5 (0–6.5)	*<0.001^d^*
*Number of relapses 2 yrs prior to baseline*				
Median (range)	2 (1–4)	1 (1–4)	2 (1–4)	*0.003^d^*
*Dominant symptom of the index relapse, n (%)*				*<0.001^b^*
Pyramidal	160 (53.3%)	65 (40.6%)	95 (67.9%)	
Sensory/pain	64 (21.3%)	49 (30.6%)	15 (10.7%)	
Visual/optic	31 (10.4%)	26 (16.3%)	5 (3.5%)	
Brainstem	24 (8%)	13 (8.1%)	11 (7.9%)	
Cerebellar	21 (7%)	7 (4.4%)	14 (10.0%)	
*Most disabled KFS, n (%)*				*0.001^b^*
Pyramidal	210 (70%)	102 (63.8%)	108 (77.1%)	
Sensory/pain	33 (11%)	19 (11.9%)	14 (10%)	
Cerebellar	23 (7.7%)	10 (6.3%)	13 (9.3%)	
Visual/optic	20 (6.7%)	18 (11.3%)	2 (1.4%)	
Others^*^	14 (4.7%)	11 (6.9%)	3 (2.1%)	
*DMT prior to baseline, n (%)*				*0.11^b^*
IFN β-1a 22 μg sc tiw	147 (49%)	75 (46.9%)	72 (51.4%)	
IFN β-1a im	55 (18.3%)	37 (21.1%)	18 (12.9%)	
Glatiramer acetate	53 (17.7%)	28 (17.5%)	25 (17.9%)	
IFN β-1bsc	45 (15%)	20 (12.5%)	25 (17.9%)	
*Duration of DMT, years*				
Median (range)	2.4 (0.1–14.3)	2.6 (0.1–14.3)	2.0 (0.1–13.4)	*0.03^c^*

*SD, standard deviation; MS, multiple sclerosis; EDSS, Expanded Disability Status Scale (27); KFS, Kurtzke Functional System (27); DMT, disease modifying treatment; IFN, interferon; sc, subcutaneous; tiw, three times weekly; im, intramuscular*.

* *Due to low frequency of brainstem, bowel/bladder and cerebral/mental category as the most disabled KFS these were grouped as “others” for the analysis*.

a *Parametric ANOVA (T-test)*;

b *chi-square*;

c *Wilcoxon test*;

d *median test*.

### Primary Endpoint Results

**Figure 1:**
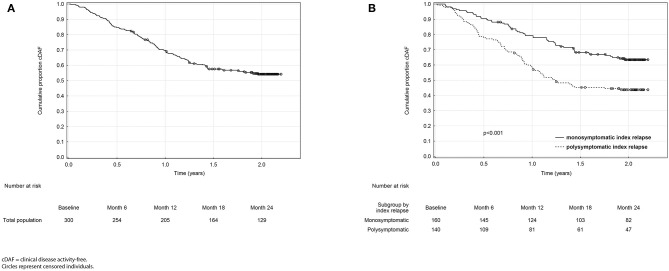
Kaplan-Meier analyses of time from baseline to clinical disease activity in the total population **(A)** and in monosymptomatic vs. polysymptomatic index relapse group **(B)**.

The overall proportion of patients remaining in the cDAF status was 84.7, 69.5, 57.5, and 54.2% at 6, 12, 18, and 24 months, respectively (Figure 1A, Table 2). At all timepoints, the cumulative probability of remaining in the cDAF status was significantly higher in the monosymptomatic index relapse group compared to the polysymptomatic index relapse group (Table 2). Cox regression hazard model showed significant difference between both index relapse groups (*p* < 0.001) with a higher hazard rate of cDAF status for patients with a monosymptomatic index relapse vs. polysymptomatic index relapse (HR = 1.94, 95% CI 1.38–2.73; Figure 1B).

**Table 2:** Primary analysis: comparison of relapse-free, disability progression-free, and cDAF status in mono- vs. polysymptomatic relapse group.

	**Total (*n =* 300)**	**Monosymptomatic relapse group (*n =* 160)**	**Polysymptomatic relapse group (*n =* 140)**	***p-value***
**Study endpoints**				
cDAF status, n (%^*^)				*<0.001^a^*
Month 6 visit	254 (84.7%)	145 (90.6%)	109 (77.9%)	
Month 12 visit	205 (69.5%)	124 (79.2%)	81 (58.5%)	
Month 18 visit	164 (57.5%)	103 (68.3%)	61 (45.3%)	
Month 24 visit	129 (54.2%)	82 (63.5%)	47 (43.7%)	
Relapse-free status, n (%^*^)				*<0.001^a^*
Month 6 visit	258 (86%)	146 (92.3%)	112 (80%)	
Month 12 visit	210 (71.2%)	128 (81.7%)	82 (59.2%)	
Month 18 visit	178 (62.6%)	109 (72.1%)	69 (51.8%)	
Month 24 visit	133 (55.9%)	83 (64.5%)	50 (46.2%)	
Disability progression-free status, n (%^*^)				*0.011^a^*
Month 6 visit	290 (96.7%)	156 (97.5%)	134 (95.7%)	
Month 12 visit	272 (93%)	147 (94.3%)	125 (91.3%)	
Month 18 visit	246 (87.8%)	138 (92.4%)	108 (82.5%)	
Month 24 visit	197 (87.8%)	110 (92.4%)	87 (82.5%)	

*cDAF, clinical disease activity-free*.

a *Log-rank test*.

* *Percentages represent the cumulative probability of survival estimated from Kaplan-Meier survival analysis censored for patients who terminated early, or whenever the endpoint had not been reached*.

### Secondary Endpoints

**Figure 2:**
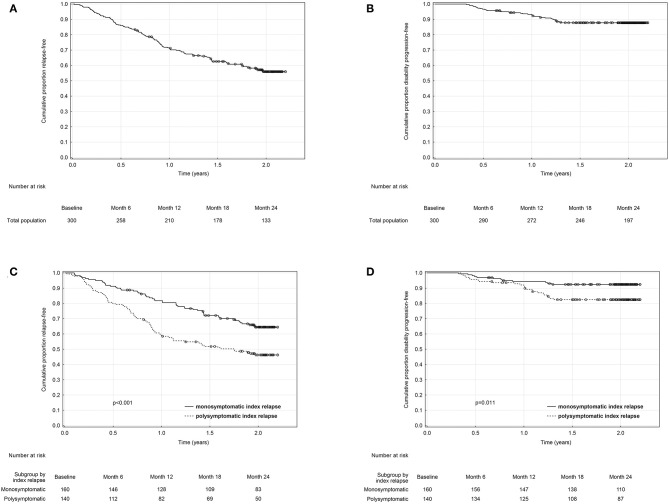
Kaplan-Meier analyses of time from baseline to first relapse and disability progression in the total **(A,B)** and in monosymptomatic vs. polysymptomatic index relapse group **(C,D)**.

The overall proportion of patients remaining relapse-free was 86, 71.2, 62.6, and 55.9% (Table 2, Figure 2A), and the overall proportion of patients remaining disability progression-free was 96.7, 93, 87.8, and 87.8% (Table 2, Figure 2B) at 6, 12, 18, and 24 months. Cumulative probability of remaining relapse-free and disability progression-free was significantly higher in patients with a monosymptomatic index relapse compared to patients with a polysymptomatic index relapse at all time points (Table 2). Likewise, time to first occurrence of a relapse (HR = 1.88, 95% CI 1.33–2.67, *p* < 0.001; Figure 2C) and disability progression (HR = 2.39, 95% CI 1.20–4.78, *p* = 0.011; Figure 2D) was significantly longer in patients with a monosymptomatic index relapse compared to patients with a polysymptomatic index relapse.

### Impact of Baseline Predictors on cDAF Status

**Figure 3:**
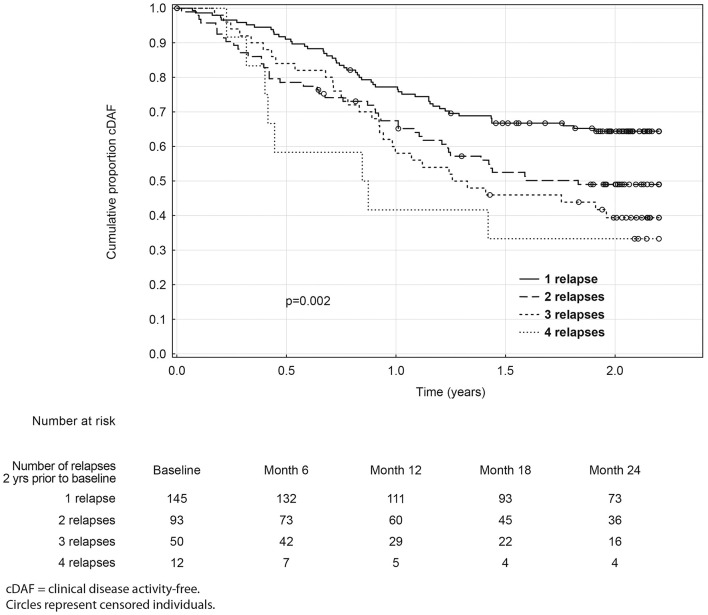
Kaplan-Meier analyses of time to first event by relapse count prior to baseline.

**Figure 4:**
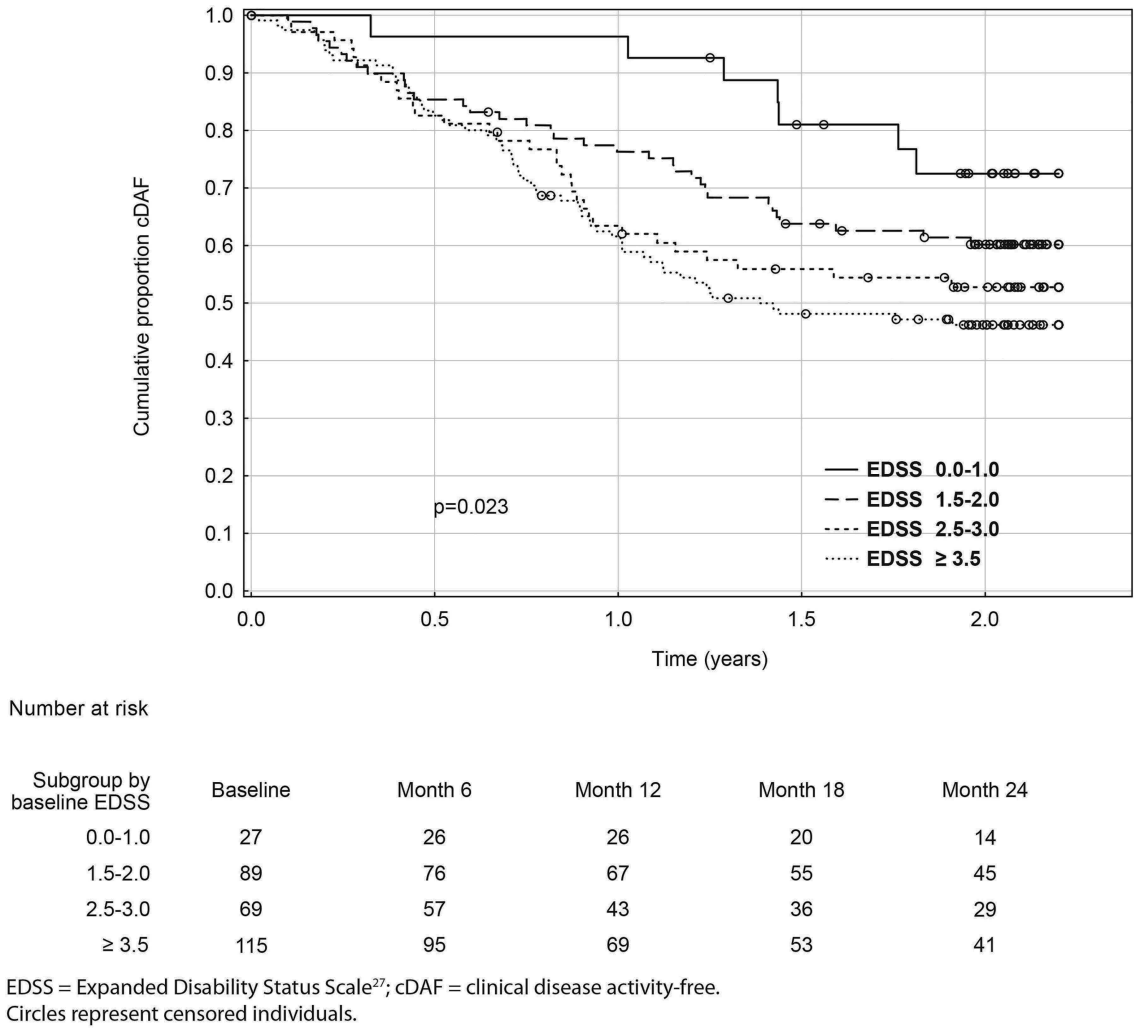
Kaplan-Meier analyses of time to first event by baseline EDSS.

Over the 2 year study period, time to clinical disease activity was significantly shorter for patients with higher number of relapses prior to baseline (*p* = 0.002; Figure 3) and for patients with higher baseline EDSS (*p* = 0.023; Figure 4, Table 3). The statistical strongest cut off for remaining in the cDAF status vs. developing a relapse and/or disability progression was 1 vs. 2–4 relapses (HR = 1.83, 95% CI 1.29–2.59), and EDSS <2.5 vs. EDSS ≥2.5 (HR = 1.65, 95% CI 1.14–2.37). Age at baseline did not have a significant impact on time to relapse and/or disability progression (*p* = 0.08 for statistically strongest cut off 30 years). However, for the factor of age at first MS symptom the cut off 25 years showed statistically significant results by both log rank test (*p* = 0.048) and Cox regression model (HR = 1.46, 95% CI 1.00–2.12). There was no statistically significant difference in time to clinical disease activity in groups by disease duration (*p* = 0.12 for statistically strongest cut off 25 years), dominant symptom of the index relapse (*p* = 0.51), the most affected KFS (*p* = 0.74), or type of DMT used prior to baseline (*p* = 0.35).

**Table 3:** Secondary analysis: comparison of cDAF status by baseline predictive factors.

	**Total, *n* (%)**	**Proportion of cDAF patients in the timepoint (%)**				***p-value***
		**Month 6**	**Month 12**	**Month 18**	**Month 24**	
**Baseline predictive factors**						
*Sex*						*0.54^a^*
Female	224	84.8	69.1	58.6	55.6	
Male	76	84.2	70.8	54.3	49.8	
*Age at first MS symptom (yrs)*						*0.26^a^*
0–19	42 (14%)	88.1	73.8	71.4	69	
20–24	61 (20.3%)	85.3	76.8	61.1	57.3	
25–29	55 (18.3%)	85.5	72.5	53.8	45.1	
30–34	47 (15.7%)	87.2	68.1	59.6	57.4	
35–39	38 (12.7%)	81.6	55.3	39.5	36.8	
40–44	32 (10.7%)	87.5	65.0	58.5	58.5	
45–49	16 (5.3%)	62.5	56.3	56.3	56.3	
50–99	9 (3%)	88.8	88.8	55.5	55.5	
*Age at baseline (yrs)*						*0.73^a^*
0–24	28 (9.3%)	85.7	78.6	67.9	67.9	
25–29	38 (12.7%)	84.2	76.1	67.9	61.7	
30–34	50 (16.7%)	86	67.7	56.9	49.5	
35–39	55 (18.3%)	81.8	67.3	52.7	49	
40–44	49 (16.3%)	87.8	67.4	46.9	44.8	
45–49	31 (10.3%)	83.9	58.1	54.8	51.2	
50–54	30 (10%)	83.3	69.8	59.4	59.4	
55–99	19 (6.3%)	84.2	78.9	68.4	68.4	
*Disease duration, yrs*						*0.12^a^*
0–1	12 (4%)	91.7	83.3	75	75	
1–2	36 (12%)	80.6	61.1	47.2	41.7	
2–3	23 (7.7%)	65.2	52.2	42.7	42.7	
3–5	50 (16.7%)	88	73.5	58.7	53.8	
5–7	33 (11%)	90.9	75.8	69.4	69.4	
7–10	59 (19.7%)	81.4	62.3	50.2	48.2	
10–15	50 (16.7%)	86	82	69.6	63.2	
15–20	24 (8%)	91.7	70.8	50	50	
20–25	9 (3%)	88.9	44.4	44.4	44.4	
25–31	4 (1.3%)	100	100	100	100	
*Number of relapses within 2 yrs prior to baseline*						*0.002^a^*
1	145 (48.3%)	91	77.2	66.7	64.4	
2	93 (31%)	78.5	67.4	52.5	49	
3	50 (16.7%)	84	58	46	39.4	
4	12 (4%)	58.3	41.7	33.3	33.3	
*Dominant symptom of the index relapse*						*0.51^a^*
Pyramidal	160 (53.3%)	81.9	66.7	53.2	51.8	
Sensory/pain	64 (21.3%)	90.6	74.8	60.1	56.6	
Visual/optic	31 (10.3%)	90.3	74.2	71	67.6	
Brainstem	24 (8%)	87.5	62.5	54.2	45.8	
Cerebellar	21 (7%)	76.2	76.2	66	54.5	
*Baseline EDSS score*						*0.023^a^*
0.0–1.0	27 (9%)	96.3	96.3	81	72.5	
1.5–2.0	89 (29.7%)	85.4	76.3	63.8	60.2	
2.5–3.0	69 (23%)	82.6	63.5	56	52.8	
≥3.5	115 (38.3%)	82.6	61.6	48.1	46.2	
*Baseline most affected KFS*						*0.74^a^*
Pyramidal	210 (70%)	83.3	67.5	54.9	52.8	
Sensory/pain	33 (11%)	87.9	72.4	62.5	59.2	
Cerebellar	23 (7.7%)	78.3	64.7	55.4	49.9	
Visual/optic	20 (6.7%)	90	75	70	64.6	
Others^*^	14 (4.7%)	100	92.9	71.4	57.1	
*DMT prior to baseline*						*0.35^a^*
IFN β-1a 22 μg sc tiw	147 (49%)	83	67.8	55.2	52.1	
IFN β-1a im	55 (18.3%)	83.6	72.5	59.4	55.1	
Glatiramer acetate	53 (17.7%)	83	66	52.8	48.9	
IFN β-1bsc	45 (15%)	93.3	75.6	68.7	66.2	

*cDAF, clinical disease activity free; EDSS, Expanded Disability Status Scale (27); KFS, Kurtzke Functional System (27); DMT, disease modifying treatment; IFN, interferon; sc, subcutaneous; tiw, three times weekly; im, intramuscular*.

* *Due to low frequency of brainstem, bowel/bladder and cerebral/mental category as the most disabled KFS, these were grouped as “others” for the analysis*.

a *Log-rank test*.

*Percentages represent the cumulative probability of survival estimated from Kaplan-Meier analysis censored for patients who terminated early, or whenever the endpoint had not been reached*.

To improve identification of risk groups tested in the univariate analyses and to uncover complex interactions between predictors of clinical disease activity, we have performed *post-hoc* multivariate analyses processed in the form of CART. These showed that, despite the statistical insignificance of KFS as a prognostic factor for cDAF for the entire group of all patients, certain factors were found to be prognostically significant in certain groups (CART branches).

**Figure 5:**
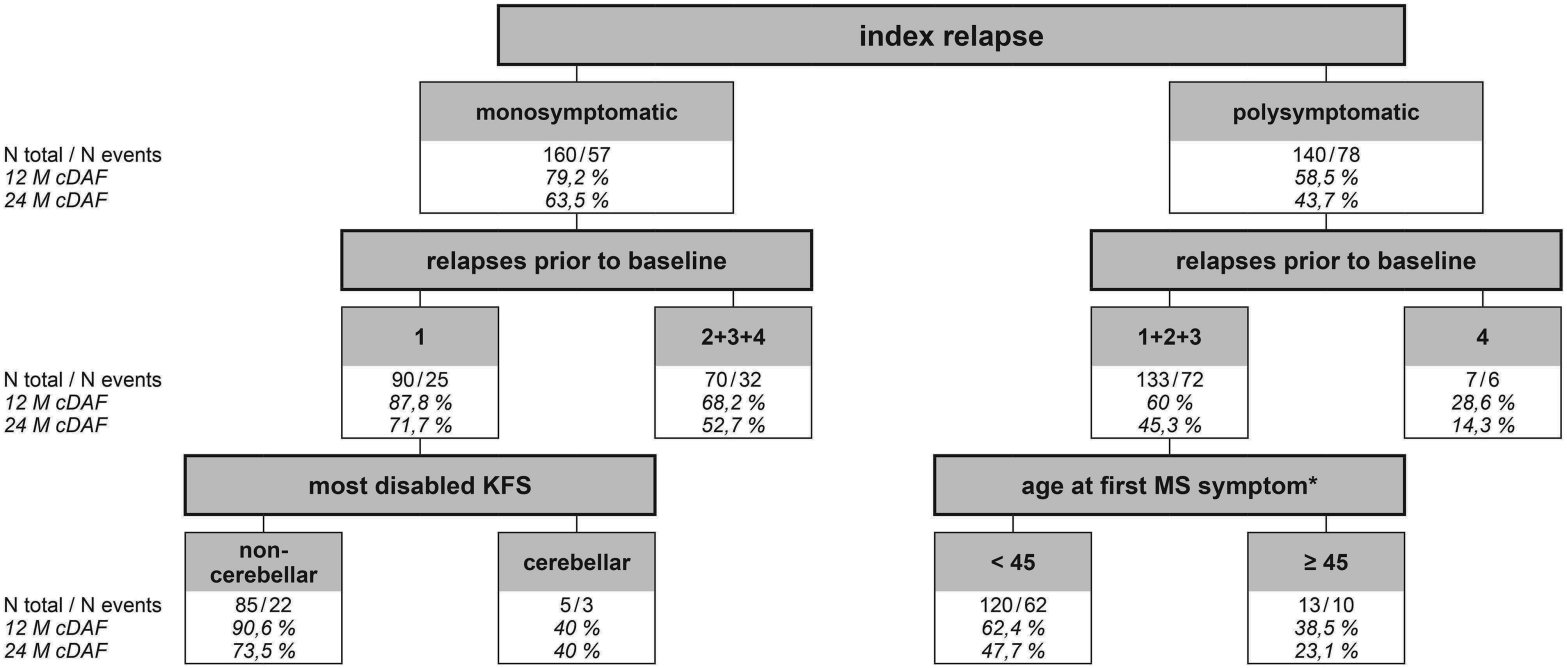
Classification & Regression Tree showing probability of cDAF status for different groups of patients. Statistically strongest factor of clinical disease activity (relapse and/or disability progression) was a polysymptomatic index replase. It divides the entire set of all patients into 2 groups monosymptomatic and polysymptomatic (first level of CART). Twelve-month cDAF shows the probability that the patient will remain without a relapse and disability progression (cDAF status) within the period. 63.5% of patients in the monosymptomatic index relapse group remained in the cDAF status at month 24, compared to 43.7% of patients in the polysymptomatic index relapse group (point 1 of CART). In the monosymptomatic group (*n* = 160, of which 57 had clinical disease activity within the study period) number of relapses prior to baseline was the strongest predictor of cDAF status (second level of CART), broken down by 1 vs. 2–4 relapses (point 2). Next statistically significant factor within the group of patients with a single monosymptomatic relapse prior to baseline was the most disabled KFS (third level of CART). Cerebellar syndrome as the most disabled KFS was at the strongest risk of clinical disease activity (point 3). In the monosymptomatic group with 2–4 relapses prior to baseline, no other statistically significant factor was found. Within the polysymptomatic group, number of relapses prior to baseline was the next statistically strongest predictor of clinical disease activity broken by 1–3 vs. 4 relapses (point 4). In the polysymptomatic of relapses group with 4 relapses prior to baseline no other statistically significant factor was found The last significant factor fond was within the polysmptomatic index relapse group with 1–3 relapses prior to baseline, which was a factor of age at first MS symptom with a cut off 45 years (point 5). cDAF, clinical disease activity-free; KFS, Kurtzke Functional System (27); MS, multiple sclerosis. *the variable “disease duration” was correlated with variable “age at first MS symptom” and was therefore used a background variable.

CART has confirmed that having a polysymptomatic index relapse was the strongest determinant of subsequent disease activity of all studied variables (Figure 5). The probability of remaining in the cDAF status at month 24 was 63.5% for patients with a monosymptomatic index relapse, compared to 43.7% for patients with a polysymptomatic index relapse (*point 1* of CART; *p* < 0.001). In the monosymptomatic index relapse group, number of relapses prior to baseline, broken down by 1 vs. 2–4 relapses, was the strongest predictor of cDAF status (second level of CART, *point 2*; *p* = 0.008). In addition, within the group of patients with a single monosymptomatic relapse prior to baseline, the next statistically significant factor was the most disabled KFS (third level of CART). Cerebellar syndrome as the most disabled KFS was at the strongest risk of a relapse or EDSS progression, while all other variants of the most disabled KFS had higher rates of cDAF than cerebellar in this point (*point 3* of CART, *p* = 0.02). In the group of patients with a monosymptomatic index relapse with 2–4 relapses prior to baseline, no other statistically significant factor was found.

Also within the polysymptomatic index relapse group, number of relapses prior to baseline was the next statistically strongest predictor of cDAF status, broken down by 1–3 vs. 4 relapses (*point 4* of CART, *p* = 0.017). However, the number of patients in the group with 4 pre-baseline relapses was small. The last significant factor found was within the polysymptomatic index relapse group with 1–3 relapses prior to baseline, and this was a factor of age at first MS symptom with cut off 45 years (*point 5* of CART, *p* = 0.014).

Classification and regression tree confirmed that the strongest predictor of clinical disease activity was having a polysymptomatic index relapse, thus confirming the correctness of our results and the assumption on which our study was designed.

### Safety/Tolerability

In total, 26 patients (8.7%) experienced 27 adverse drug reactions (ADRs), all in line with the well-characterized sc IFN β-1a safety profile. The most common ADRs were flu-like symptoms (5%; *n* = 15), injection site reactions (2.7%; *n* = 8), hair loss (0.3%; *n* = 1), depression (0.3%; *n* = 1), pruritus (0.3%; *n* = 1), and liver enzyme elevation (0.3%; *n* = 1). All ADRs were classified as non-serious; 9 of them were mild, 15 moderate, and 3 severe. Twelve ADRs led to a discontinuation of the study medication, 4 led to a reduction of the study medication dose, 7 required other treatment, and 4 cases did not require any intervention. In six patients (2%), 7 cases of maternal exposure to the drug during pregnancy have been reported and the treatment had been discontinued. Five of the pregnancies came to a healthy live birth, 2 consequent pregnancies in one patient were terminated by a spontaneous abortion and were reported as a serious adverse event. No death has been reported in the study.

## Discussion

In this observational study we have analyzed the relationship between clinical factors and future clinical disease activity, in spite of treatment change to higher dose of IFN β-1a, often used as an active comparator in clinical trials (33, 34). We have confirmed that the risk of clinical disease activity was significantly higher in the polysymptomatic (vs. monosymptomatic) index relapse group and that the polysymptomatic index relapse was the strongest and independent determinant of subsequent disease activity of all studied variables. Nowadays, due to availability of newer high-efficacy therapies, changing from one injectable platform therapy to another injectable platform treatment due to suboptimal response (e.g., relapses) is at least questionable. However, there are still situations when such change is considered in a clinical practice, because either individual factors (e.g., safety, pregnancy planning, patient's preference, economics reason etc.) or local market factors (e.g., reimbursement of a suitable high-efficacy drug restricted or not granted, limited availability on a market etc.). Then, our results may help when making a treatment decision.

Polysymptomatic relapse phenotype at MS onset is a known factor of unfavorable prognosis (25, 35–42), however, not many studies have focused on this relapse characteristic later in the disease course. The original study by Runmarker and Andersen performed before the era of DMTs demonstrated that the occurrence of polyregional symptoms at the last relapse remained a significant factor of unfavorable prognosis not only during the first 5 years of disease, but also for the long-term (25). Our study is the first one to confirm these findings in the setting of 44 μg IFN β-1a as an therapeutic option following other platform injectables, further confirming the prognostic importance of the polysymptomatic relapse phenotype.

The second statistically significant predictor of post-baseline clinical disease activity in our study was the number of relapses prior to baseline. This finding has been reported in some previous studies of IFN β (43–45), but was not confirmed according to other studies (46–48). These conflicting results may arise mainly from a different definition of treatment response, different length of follow-up, and variability between baseline disease activity and treatment duration in these studies (43–48). The fact that even the same studies reported different conclusions at different times suggests methodological rather than intrinsic variability.

Several studies have found an association between higher baseline EDSS score and lack of response to IFN β (44, 46, 49–51), however, higher early EDSS score can also be considered a marker of an adverse prognosis, regardless of the disease-modifying agent received. In this respect, our study showed significantly shorter time to clinical disease activity for patients with higher baseline EDSS. Considering the wide variability of baseline EDSS in our study, involving also higher scores, we cannot exclude that some of these patients may have already entered the overlapping phase between the late relapsing-remitting and early progressive phase with ongoing relapses. Secondary progressive MS is a retrospective diagnosis, defined as insidious and irreversible worsening of neurological function lasting for ≥ 1 year (52), following the relapsing-remitting course, and being independent of relapse-related worsening (53). Only 2% of patients reach sustained disability level of EDSS 6 (needing unilateral gait support) or above in the RRMS phase (53–55), indicating that 98% of disability of EDSS 6 is driven by the progressive disease course, which is the strongest determinant of poor long-term prognosis in MS (25, 52–61). Relapses in MS are generally considered self-limiting, tending to diminish over time (either with increasing age or longer disease duration) (62–68). A shorter time to clinical disease activity in the group of patients with the highest EDSS scores might therefore had been caused by post-baseline EDSS progression due to overlapping early progressive phase rather than by relapses.

Impact of IFN β on clinical disease activity in RRMS could be considered at three main levels: decrease of relapse rate, absence of disability progression, and absence of conversion to secondary-progressive phase. These factors are inter-related, since disability progression in MS results from insufficient recovery from relapses and/or progressive disease course. In our study, the future disease activity after treatment change to higher-dose IFN β-1a was measured by the absence of relapses and the absence of sustained disability progression. After 2 years of follow-up, more than half of the patients remained relapse-free and a vast majority of patients had no sustained disability progression, thus the clinical disease break through activity in these patients was caused mainly by relapse-associated temporary EDSS changes, rather than by sustained progression of disability.

The CART analyses further showed that within the polysymptomatic relapse group with <4 relapses prior to baseline, patients above the age of 45 at first MS symptom are exposed to a higher risk of post-baseline clinical disease activity. The lower number of patients in the group with 4 pre-baseline relapses could impact the relatively smaller effect of this group on post-baseline clinical disease activity compared to the group with 1–3 relapses. An older age at MS onset (>40 years) is a generally known unfavorable prognostic factor. Age is a critical determinant of recovery from MS relapses (25, 62, 69–75) and the probability of complete recovery from a relapse decreases by ~1% per year after the index event (74). Both factors responsible for sustained disability progression in MS (relapse recovery and progressive MS onset) are age-related. Prior studies have demonstrated that relapse-recovery declines with age in a linear fashion (70), unlike the onset of progressive disease phase triggering a shift around the age of 45 (53, 54, 57, 58, 60). These associations could be mediated through a number of mechanisms related to the complex interplay between injury and repair and change to neurodegenerative phenotype in MS.

A study by Waubant et al. (46) found a correlation of an older age at MS onset with better response to IFN β, however, the patients in this study also tended to have a longer disease duration and might represent a subset of MS patients with milder disease. Furthermore, the best multivariable model used in their study predicted response correctly only in 73% of cases (40). A study by Fromont et al. (48) found a similar correlation of older age at MS onset with better response to IFN β, but only for a criterion of lower annual relapse rate under IFN β than during the year preceding treatment and not when compared to the 2 years prior to treatment, or for a criterion of any relapse on IFN β or progression of disability. This study therefore concluded that only the relapse-rate in the year before initiation of IFN β was able to predict the treatment response (48).

Besides polysymptomatic disease, relapse frequency and age at onset, the long-term disability accrual in MS is also associated with relapse phenotype, of which pyramidal and cerebellar relapses pose a relatively higher risk of incomplete recovery (69, 76, 77). Cerebellar relapses also seem to become more frequent later in the disease course or in older patients (76), further increasing the risk of irreversible disability. In the CART model we presented, cerebellar system as the most disabled KFS in patients with only single monosymptomatic relapse prior to baseline was associated with a higher risk of clinical disease activity.

Relapse location also seems to interact with relapse fulminance and precondition the patient to limited remyelination and progressive axonal degeneration (69). Furthermore, previous studies have shown that patients tend to experience relapses, which are phenotypically similar to their preceding clinical episodes (63, 78, 79) and pathology findings confirmed that areas of demyelination are commonly localized within previously remyelinated regions (64, 80). Therefore even a single relapse with cerebellar involvement can precondition an individual to further disability accrual.

Outcome measures based on relapses have some limitations. As mentioned above, in MS there is a spontaneous reduction in the number of relapses over time (65–68, 81–83), when axonal degeneration predominates over inflammation. The impact of symptomatic attacks on disability also seems to decrease with time (84). Nevertheless, analyses of disability progression as our other outcome measure showed even more statistically significant results to support our findings.

Another limitation of our study is inherent to EDSS as a measure of neurological disability. The established KFS and EDSS steps to measure disability in MS are not even and are not linear. EDSS has a bias toward ambulation and a ceiling effect, since impairment in the KFS cannot raise the EDSS above 4.0 unless ambulatory impairment is also present. Despite that, EDSS is of enduring value and remains the gold standard for observing disability progression in controlled trials. In our study, the disability progression was confirmed if a patient's EDDS increased by at least 1.0 point compared to the baseline. Depending on a baseline EDSS, 1.0 point change in EDSS could correspond to patient losing the ability of unrestricted gait.

Because of the nature of an observational study, there was no placebo or therapeutic comparator group, therefore our results do not provide comparative efficacy data. Patient selection was not randomized, but vast majority of eligible patients from each MS center were included in the study.

The duration of DMT prior to treatment change was significantly longer in the monosymptomatic index relapse group. It is possible, that patients with less severe relapses remained longer on their prior treatment due to limited options of treatment escalation at the time of enrollment. In 2012–2013 when our study was conducted, fingolimod and natalizumab were the only options available for treatment escalation for patients who developed a minimum of 2 severe relapses per year or 3 severe relapses per 2 years during treatment with first-line DMTs (low-dose IFN β-1a, IFN β-1b, or glatiramer acetate). Reimbursement for fingolimod in the Czech Republic was not granted until the beginning of 2013. In patients with higher EDSS scores and more aggressive forms of MS, early initiation or treatment change to higher-dose IFN β-1a (44 μg) was recommended per local treatment guidelines. Therefore, it is possible, that more active patients could had been potentially escalated to natalizumab or fingolimod rather than to 44 μg IFN β-1a and this may have introduced selection bias. Our results therefore should be applied to population of patients who did not meet the above described criteria for escalation to natalizumab or fingolimod. 22.3% of patients enrolled in our study were lost to follow up, out of these 13% terminated early for insufficient efficacy and were recorded as having clinical disease activity, i.e., they were not censored and could not have been a source of bias. The rest (9.3%) of patients terminated early for various reasons, out of these 2,7% of patients with voluntary withdrawal continued to use 44 μg dose of IFN β-1a but were not further followed in the study. They could have been a source of bias if they had a post-baseline clinical disease activity by the end of the study period. However, at the rate of 2.7% the risk of selection bias is low. Patients who had a moderate or severe relapse during the study and have met the reimbursement criteria for fingolimod or natalizumab were escalated and could be a source of retention bias. Some unmeasured confounding (e.g., adherence to treatment, treatment with immunosuppressive agents in the period more than 3 months prior to baseline) is possible in a real-world study based on healthcare data utilization.

It is worth mentioning that a higher proportion of patients in the monosymptomatic relapse group was receiving im IFN β-1a 30 μg im once weekly (21.1 vs. 12.9%), and a higher proportion of patient in the polysymptomatic relapse group were treated with sc IFN β-1b 250 μg every other day (17.9 vs. 12.5%). It is important to take this into consideration when interpreting the results.

We did not include the development of neutralizing antibodies against IFNs (NAbs), which can influence the disease activity in patients treated by different types of IFNs (85–88). However, we did not observe any significant difference when comparing different pre-baseline DMTs and occurrence of clinical disease activity after baseline. Moreover, individual genetic background that underlies the heterogeneity of MS disease and heterogeneity of treatment response should be considered. Because of high variability of MRI techniques and protocols between the medical facilities, our study did not include MRI measures of disease activity. Short of such paraclinical markers, we can conclude that patients with a polysymptomatic relapse and/or higher number of relapses in the 2 years prior to baseline are at high risk of clinical disease activity aftertreatment change to higher-dose IFN β-1a. Therefore, these patients should receive a more effective escalation therapy (such as cladribine, fingolimod or monoclonal antibodies), in order to minimize the risk of future disease activity.

## Data Availability Statement

The datasets generated for this study are available on request to the corresponding author.

## Ethics Statement

The studies involving human participants were reviewed and approved by Independent Ethics Committee NZZ Clintrial s.r.o., Prague, Czech Republic. The patients/participants provided their written informed consent to participate in this study.

## Author Contributions

MN interpreted data and wrote the manuscript. AT and JM participated in study design and data interpretation. JM was the principal investigator of the study for the Czech Republic. All authors contributed to the article and approved the submitted version.

## Conflict of Interest

MN received consultant fees from Roche, as well as research support from the European Regional Development Fund (FNUSA-ICRC CZ.1.05/1.1.00/02.0123), European Social Fund, and the State Budget of the Czech Republic. AT is an employee of Merck spol. s r.o., Prague, the Czech Republic, a local affiliate of Merck KGaA, Darmstadt, Germany. JM received presentation fees from Merck, Biogen, Sanofi-Genzyme, Roche.
